# Rocaglamide promotes the infiltration and antitumor immunity of NK cells by activating cGAS-STING signaling in non-small cell lung cancer

**DOI:** 10.7150/ijbs.65019

**Published:** 2022-01-01

**Authors:** Xuewei Yan, Chao Yao, Cheng Fang, Min Han, Chenyuan Gong, Dan Hu, Weiming Shen, Lixin Wang, Suyun Li, Shiguo Zhu

**Affiliations:** 1 Center for Traditional Chinese Medicine and Immunology Research; School of Basic Medical Sciences, Shanghai University of Traditional Chinese Medicine, 1200 Cai Lun Rd. Shanghai 201203, P. R. China.; 2 Department of Immunology and Pathogenic Biology, School of Basic Medical Sciences, Shanghai University of Traditional Chinese Medicine, 1200 Cai Lun Rd. Shanghai 201203, P. R. China.; 3 School of Acupuncture, Moxibustion and Tuina, Shanghai University of Traditional Chinese Medicine, 1200 Cai Lun Rd. Shanghai 201203, P. R. China.; 4 Department of Pathology, School of Basic Medical Sciences, Shanghai University of Traditional Chinese Medicine, 1200 Cai Lun Rd. Shanghai 201203, P. R. China.

**Keywords:** Rocaglamide, NK cells, Non-small cell lung cancer, STING, Mitochondrial DNA

## Abstract

**Background:** Natural killer (NK) cell-based immunotherapy is clinically limited due to insufficient tumor infiltration in solid tumors. We have previously found that the natural product rocaglamide (RocA) can enhance NK cell-mediated killing of non-small cell lung cancer (NSCLC) cells by inhibiting autophagy, and autophagic inhibition has been shown to increase NK cell tumor infiltration in melanoma. Therefore, we hypothesized that RocA could increase NK cell infiltration in NSCLC by autophagy inhibition.

**Methods:** Flow cytometry, RNA-sequencing, real-time PCR, Western blotting analysis, and xenograft tumor model were utilized to assess the infiltration of NK cells and the underlying mechanism.

**Results:** RocA significantly increased the infiltration of NK cells and the expressions of CCL5 and CXCL10 in NSCLC cells, which could not be reversed by the inhibitions of autophagy/ULK1, JNK and NF-κB. However, such up-regulation could be suppressed by the inhibitions of TKB1 and STING. Furthermore, RocA dramatically activated the cGAS (cyclic GMP-AMP synthase)-STING (stimulator of interferon genes) signaling pathway, and the inhibition/depletion of STING ablated the up-regulation of CCL5 and CXCL10, NK cell infiltration, and tumor regression induced by RocA. Besides, RocA damaged mitochondrial DNA (mtDNA) and promoted the cytoplasmic release of mtDNA. The mPTP inhibitor cyclosporin A could reverse RocA-induced cytoplasmic release of mtDNA.

**Conclusions:** RocA could promote NK cell infiltration by activating cGAS-STING signaling via targeting mtDNA, but not by inhibiting autophagy. Taken together, our current findings suggested that RocA was a potent cGAS-STING agonist and had a promising potential in cancer immunotherapy, especially in NK cell-based immunotherapy.

## Introduction

As a member of the group I innate lymphoid cells (ILCs), natural killer (NK) cells play a crucial role in tumor immune surveillance 1. Different from specific T cells, NK cells recognize and kill target cells without prior sensitization and restriction to major histocompatibility complex (MHC) and antigen. Due to such unique ability, NK cell-based immunotherapy has been boosted to treat cancers 2. However, the therapeutic efficacy of NK cells is clinically limited due to the poor infiltration in solid tumors 3. Therefore, it is a promising strategy to improve the antitumor immunity of NK cells by promoting NK cell homing and infiltration into tumors 4, 5.

Chemokines function as a 'zip code' for immune cell migration. NK cell homing and infiltration into tumors mainly depend on chemokines secreted by cancer cells 6. CXCL10 has been shown to play an important role in directing the homing and infiltration of NK cells into solid tumors and enhancing the NK cell antitumor efficacy 7. CCL5 is up-regulated in melanoma by autophagic inhibition, and it can promote tumor infiltration and antitumor immunity of NK cells 8. These findings suggest that it is an effective strategy to attract NK cells and enhance their antitumor immunity by priming tumor cells to secrete CXCL10 and CCL5.

Mitochondria are important bioenergetic and biosynthetic organelles. Besides their conventional role as the cellular power station, mitochondria are also critical for redox homeostasis, oncogenic signaling, innate immunity, and apoptosis 9. Mitochondrial functions are usually up-regulated in cancer cells to meet their increased metabolic demand, contributing to chemoresistance and regulating cell death pathways 10. It is well known that mitochondria play a critical role in cancer progression and cancer immune evasion through macromolecular synthesis and energy production 11, 12. Targeting mitochondria has become a novel and conceptual strategy for cancer therapy 13.

Rocaglamide (RocA), derived from Aglaia odorata, has been shown to inhibit tumor growth and sensitize TRAIL-mediated apoptosis in several cancers 14-19. In our previous study, we have found that RocA enhances NK cell-mediated killing of non-small cell lung cancer (NSCLC) cells by inhibiting autophagy via targeting autophagy initial gene ULK1 (unc-51-like kinase 1) 20. In the present study, we found that RocA damaged mitochondrial DNA (mtDNA) and promoted the cytoplasmic release of mtDNA, leading to the activation of cGAS (cyclic GMP-AMP synthase)-STING (stimulator of interferon genes) signaling pathway and increased NK cell infiltration in NSCLC. Collectively, our findings suggested that RocA was a potent cGAS-STING agonist and had a potential application in cancer immunotherapy, especially in NK cell-based immunotherapy.

## Methods

### Reagents

RocA (BBP00609) was purchased from BioBioPha Co., Ltd. (Yunnan, China). Chloroquine (CQ) (C6628), SP600125 (JNK inhibitor) (S5567), and SB202190 (p38 inhibitor) (S7067) were obtained from Sigma Aldrich. Anti-ULK1 antibody (sc-33182) was supplied by Santa Cruz Biotechnology. Alexa Fluor 647-AffiniPure Donkey Anti-Goat IgG (H+L) (705-605-003) was provided by Jackson. RBC lysis buffer (10×) (420301), PE anti-mouse CD3 (100205), PE-IgG Fc (409304), APC anti-mouse NKp46 (137608), and APC-IgG Fc (409306) were purchased from BioLegend Inc. Antibodies against β-Actin (4970), TBK1 (3504), phospho-TBK1 (Ser172) (5483), IRF3 (4302), phospho-IRF3 (Ser396) (29047), NF-κB p65(8242), and phospho-NF-κB p65 (Ser536) (3033) were obtained from Cell Signaling Technology. Antibodies against STING/TMEM173 (A3575) and cGAS (A8335) were supplied by ABclonal. Trizol (15596018), nuclease-free water (AM9938), and Lipofectamine™ 3000 (L3000015) were provided by Thermo Fisher Scientific. Goat antimouse NKp46/NCR1 antibody (AF2225) and human CCL5/RANTES ELISA kit (DY278) were purchased from R&D Systems. Collagenase I (40507ES60), DNase I (10607ES15), RIPA protein lysate (20101ES60), and BCA protein reagent assay kit (20201ES76) were obtained from Yeasen. Paraformaldehyde (4%, P0099), DAPI (4′,6-diamidino-2-phenylindole) (C1002), and BAY11-7082 (NF-κB inhibitor) (SF0011) were supplied by Beyotime. Mouse anti-human γ-H2AX antibody (Ab26350) and goat anti-mouse Alexa Fluro 488-FITC antibody (Ab150113) were purchased from Abcam. CYT387 (TBK1 inhibitor) (HY-10961), H-151 (hSTING inhibitor) (HY-112693), and C-176 (mSTING inhibitor) (HY-112906) were obtained from MCE (MedChemExpress).

### Cell culture

Human NSCLC cell lines (A549, H1299, and H1975) and mouse Lewis lung cancer (LLC) cells were obtained from the Cell Bank of Shanghai Institutes for Biological Sciences, Chinese Academy of Sciences. A549 and LLC cells were maintained in high-glucose DMEM (HyClone, SH30022.01B), and H1299 and H1975 cells were maintained in RPMI-1640 medium (HyClone, SH30809.01) supplemented with 10% fetal bovine serum (FBS, Gibco) and 1% penicillin-streptomycin (Yeasen, 60162ES76) at 37 °C in a humidified atmosphere containing 5% CO_2_.

### Subcutaneous xenograft tumor models

All animal procedures, including tumor transplantation, tumor volume monitoring, and euthanasia, were approved by the Institutional Animal Care and Use Committee at Shanghai University of Traditional Chinese Medicine. Male C57BL/6 mice (6 weeks old) were purchased from Vital River Laboratory Animal Technology Co. (Beijing, China) and maintained in a specific pathogen-free (SPF) environment. Mice were anesthetized with isoflurane. A total of 1 × 10^6^ STING^WT^ or STING^KO^ LLC cells per mouse were subcutaneously inoculated onto the upper back of C57BL/6 mice, and 1.0 mg/kg of RocA was administered via intraperitoneal (i.p.) injection every 2 days from day 3. Tumor volume was measured every 2 days and calculated using the formula as follows: V=(π/8)a×b^2^, where V=tumor volume, a=maximum tumor diameter, and b=minimum tumor diameter. Mice were humanely sacrificed by CO_2_ suffocation on day 18, and tumors were excised, weighed, photographed, and then used to detect NK cells by flow cytometry.

### NK cell migration assay

Human peripheral blood mononuclear cells (PBMCs) were obtained from the Shanghai Blood Center according to the research protocol approved by the Shanghai Blood Administration. Fresh or frozen PBMCs and irradiated mbIL-21-CD137L-K562 cells were co-cultured in RPMI-1640 complete medium for 2 weeks under the stimulation of IL2 to expand NK cells as previously described 21. NSCLC cells were treated with different concentrations (0, 12.5, and 25 nM) of RocA for 24 h, and then seeded into the bottom compartment of a transwell permeable chamber (3-μm pore size). A total of 1 × 10^5^ NK cells per well were seeded into the upper compartment. After 2 h, NK cells in the bottom chamber were collected and analyzed by flow cytometry.

### Immunofluorescence (IF) staining

Frozen sections of tumor tissue were permeabilized with the mixture of PBS: methanol: H_2_O_2_ at a ratio of 5:4:1 for 30 min and then treated with 0.2% Triton X-100 for 20 min, followed by incubation with the anti-NKp46 antibody at 4°C overnight. The tumor tissues were then exposed to Cy3-AffiniPure anti-goat antibody for 1 h and then stained with DAPI for 10 min. For the DNA damage assay, a total of 2×10^5^ A549, H1299, or H1975 cells were seeded into 6-cm glass bottom dishes, followed by exposure to 25 nM of RocA or 5 µM of camptothecin (CPT) for 24 h. Cells were fixed with 4% paraformaldehyde for 30 min and then treated with 0.2% Triton X-100 for 20 min, followed by incubation with the anti-H2AX antibody at 4°C overnight. The cells were then exposed to Alexa Fluro 488 anti-mouse antibody for 1 h, followed by incubation with DAPI for 10 min. Images were captured by laser confocal microscopy (ZEISS, Oberkochen, Germany).

### Flow cytometry analysis

Cells were incubated with appropriate fluorescence-conjugated antibodies for 30 min at 4°C in the dark, then washed and resuspended in PBS containing 1% FBS. The data were acquired by a BD Accuri C6 (BD Biosciences) and analyzed by FlowJo software.

### RNA sequencing

A total of 1×10^6^ LLC cells per mouse were subcutaneously inoculated onto the upper back of C57BL/6 mice, and 1.0 mg/kg of RocA was administered via i.p. injection every 2 days. Mice were sacrificed on day 21, and tumors were isolated and analyzed by RNA-Seq, which was performed by Shanghai Biotechnology Corporation. Genes with fold change ≥2 and P <0 .05 were identified as differentially expressed genes (DEGs).

### Real-time PCR

Total RNA was extracted from A549, H1299, H1975, LLC cells, and tumor tissues by Trizol reagent, and then reversely transcribed into cDNA by using the PrimeScriptTM RT MasterMix kit (TaKaRa, RR036A). Real-time PCR was performed on an ABI system (Applied Biosystems, Thermo Fisher Scientific Inc., MA, USA) by using a SYBR® Premix Ex Taq™ kit (TaKaRa, RR420A) according to the manufacturer's instructions. Briefly, after an initial denaturation step at 95°C for 30 s, the amplifications were carried out with 40 cycles at a melting temperature of 95°C for 5 s and an annealing temperature of 60°C for 30 s, followed by melt curve analysis. The relative expressions of the target genes were calculated by using the 2^-ΔΔ^Ct method. Primers were listed as follows: human CCL5 forward: TTGCCTGTTTCTGCTTGCTC; human CCL5 reverse: TGTAACTGCTGCTGTGTGGT; human CXCL10 forward: AACCTCCAGTCTCAGCACCATGAA; human CXCL10 reverse: AGGTACAGCGTACAGTTCTAGAGAG; human STING forward: AGCATTACAACAACCTGCTACG; human STING reverse: GTTGGGGTCAGCCATACTCAG; human TBK1 forward: TGGGTGGAATGAATCATCTACGA; human TBK1 reverse: GCTGCACCAAAATCTGTGAGT; human IRF3 forward: AGAGGCTCGTGATGGTCAAG; human IRF3 reverse: AGGTCCACAGTATTCTCCAGG; human NF-κB1 forward: AACAGAGAGGATTTCGTTTCCG; human NF-κB1 reverse: TTTGACCTGAGGGTAAGACTTCT; human 18S forward: GTAACCCGTTGAACCCCATT; human 18S reverse: CCATCCAATCGGTAGTAGCG; mouse CCL5 forward: CTGCTGCTTTGCCTACCTCT; mouse CCL5 reverse: CGAGTGACAAACACGACTGC; mouse CXCL10 forward: AGGGGAGTGATGGAGAGAG; mouse CXCL10 reverse: TGAAAGCGTTTAGCCAAAAAAGG; mouse IFN-γ forward: AGTGGCATAGATGTGGAAGAAAAGA; mouse IFN-γ reverse: TCAGGTGTGATTCAATGACGCTTAT; mouse 18S forward: GTAACCCGTTGAACCCCATT; mouse 18S reverse: CCATCCAATCGGTAGTAGCG.

### Western blotting analysis

A total of 3×10^5^ A549, H1299, or H1975 cells were seeded into 6-cm plates and exposed to 25 nM of RocA for 1, 3, 6, 12, and 24 h. LLC cells were exposed to different concentrations (0, 25, and 50 nM) of RocA for 24 h. The cells were lysed with RIPA buffer containing protease and phosphatase inhibitors. Protein contents were quantified by a BCA protein reagent kit. Equal amounts of proteins were subjected to sodium dodecyl sulfate-polyacrylamide gel electrophoresis (SDS-PAGE) using 8~10% gels, followed by immunoblotting using an enhanced chemiluminescence substrate (MerckMillipore, WBKLS0500). Immunoreactive bands were automatically visualized by using a chemiluminescent detection system (ProteinSimple, CA, USA).

### ELISA (enzyme-linked immunosorbent assay)

A total of 3×10^5^ A549, H1299, or H1975 cells were seeded into 6-cm plates and incubated with different concentrations (0, 12.5, and 25 nM) of RocA for 24 h. The expression of CCL5 was analyzed by using a human CCL5 ELISA Kit (R&D Systems) according to the manufacturer's instructions. The plates were read using a spectrophotometer (BioTek) at an excitation wavelength of 450 nm and an emission wavelength of 570 nm.

### mtDNA cytoplasmic release assay

Real-time PCR was used to determine the mtDNA release in the cytoplasm. A total of 3×10^5^ A549, H1299, or H1975 cells were seeded into 6-cm dishes and exposed to different concentrations (0, 25, 50, and 100 nM) of RocA or different concentrations (0, 6.25, and 12.5 µM) of cyclosporin A (CsA) for 12 or 24 h. The cells were lysed with 0.1% NP-40, incubated on ice for 15 min, and then centrifuged at 13,000 rpm for 15 min at 4 °C. The cytoplasmic mtDNA was purified by using a DNeasy Blood and Tissue Kit (Qiagen, 69504), and then real-time PCR was performed by using a SYBR® Premix Ex Taq™ kit on an ABI system. The relative expressions of target genes were calculated using the 2^-ΔΔ^Ct method. Primers were listed as follows: human cytochrome c oxidase I (CO1) forward: CAGGAGTAGGAGAGAGGGAGGTAAG; human CO1 reverse: TACCCATCATAATCGGAGGCTTTGG; human mtND1 forward: CACCCAAGAACAGGGTTTGT; human mtND1 reverse: TGGCCATGGGTATGTTGTTAA; human D-Loop forward: CTATCACCCTATTAACCACTCA; human D-Loop reverse: TTCGCCTGTAATATTGAACGTA; human RPL13A forward CGCCCTACGACAAGAAAAAG; human RPL13A reverse CCGTAGCCTCATGAGCTGTT.

### RNA interference assay

Small interfering RNA (siRNA) was synthesized from Shanghai Jima Biological Co., Ltd. and transfected into NSCLC cells by Lipofectamine 3000. After 48 h, cells were collected. The siRNA sequence for human STING was 5′-GCAUUACAACAACCUGCUATT-3′. The negative control sequence was 5′-UUCUCCGAACGUGUCACGU-3′.

### STING depletion assay

A CRISPR/Cas9 system was used for the STING depletion assay. The mouse STING guiding RNA sequence was 5′-ACCGGTCCAAGTTCGTGCGAGGCT-3′. The guiding RNA oligonucleotide was inserted into pGL3-U6-SgRNA-PGK-puromycin vector and then transfected into LLC cells by Lipofectamine 3000. After 24 h, cells were diluted and seeded into 96-well plates for clone growth. After 2 weeks, single colonies were picked up, and the expression of STING was determined by Western blotting analysis.

### Statistical analysis

All data were analyzed by using GraphPad Prism version 7 and expressed as means ± standard deviation (S.D.). A two-sided Student's t-test was used to compare the means of individual treatments, and a one-way ANOVA was carried out to compare the means of three or more treatments. A P value <0.05 was considered statistically significant.

## Results

### RocA promotes the infiltration of NK cells

**Figure 1:**
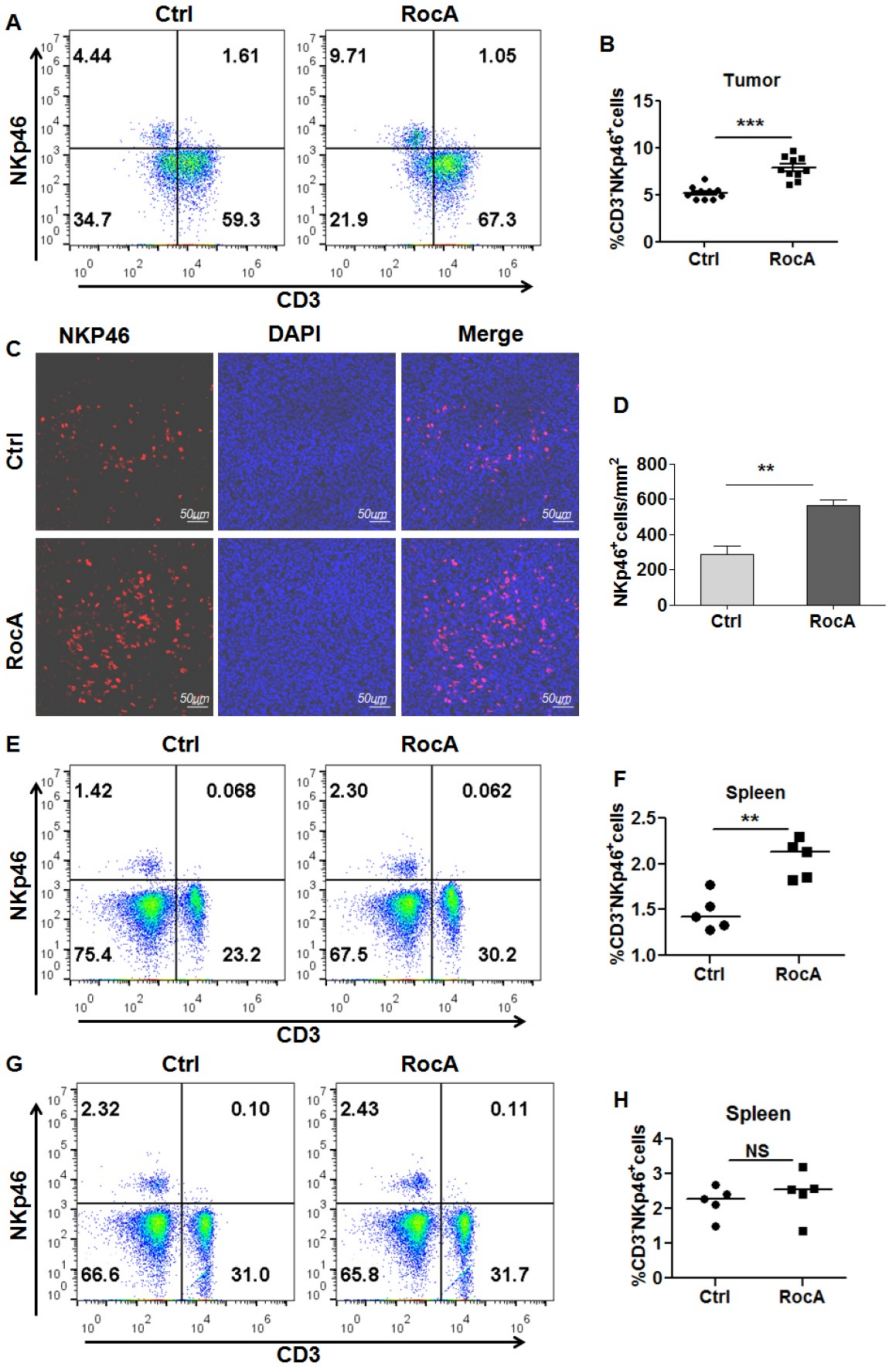
** RocA increases NK cell infiltration in NSCLC.** LLC cells were subcutaneously inoculated onto the upper back of C57BL6 mice on day 0, and 1 mg/kg of RocA was administered by i.p. injection every 2 days from day 3. Mice were sacrificed on day 21, and tumors were excised. The proportion of NK cells in tumors were analyzed by flow-cytometry **(A-B)**. Tumor tissues were stained with anti-NKp46 antibody and DAPI **(C)**, and the NKp46^+^ NK cells were quantified in tumor tissues **(D)**. The proportion of NK cells in spleens in LLC tumor-bearing mice **(E)** and normal mice without LLC tumor **(F)** were analyzed by flow-cytometry.

Since RocA enhances NK cell-mediated killing of NSCLC cells by inhibiting autophagy, and autophagy inhibition can increase the infiltration of NK cells in melanoma. These findings lead us to investigate whether RocA could increase the infiltration of NK cells in NSCLC. To address this issue, LLC cell-bearing mice were treated with DMSO or RocA, and tumor tissues were isolated and used to analyze NK cells. The flow cytometry showed that RocA significantly increased the proportion of NK cells (Fig. 1A-B), and IF staining showed that RocA significantly increased the infiltration of NK cells in LLC tumor tissues (Fig. 1C-D). Additionally, RocA could increase the proportion of NK cells in the spleen in LLC cell-bearing mice (Fig. 1E-F), but not the normal mice without tumor (Fig. 1G-H). These results demonstrated that RocA could increase NK cells homing to the tumor in NSCLC. To further confirm this effect, A549, H1299, and H1975 cells were treated with RocA and the migration of NK cells was then investigated* in vitro*. Results showed that RocA significantly promoted NK cells migrating to A549, H1299, and H1975 cells in a dose-dependent manner (Fig. S1A-C). Taken together, these findings indicated that RocA could promote the infiltration of NK cells in NSCLC.

### RocA increases the expression of CCL5 in NSCLC cells independent of autophagy inhibition

**Figure 2:**
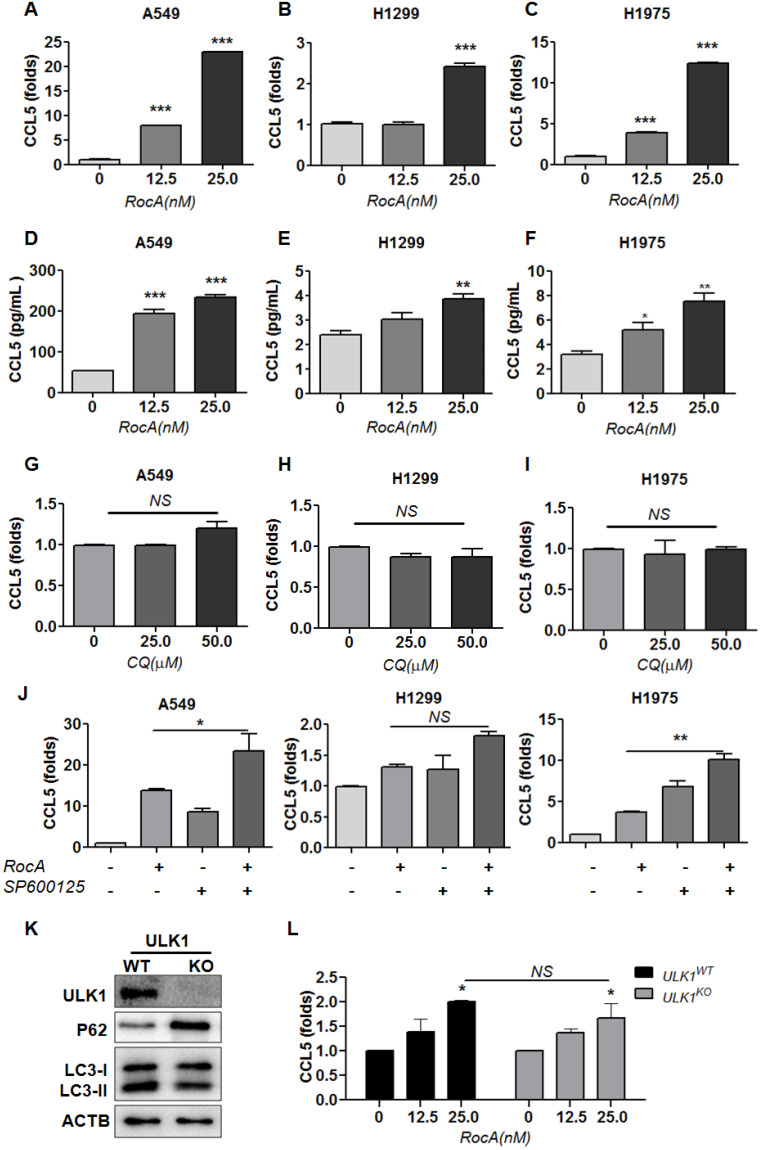
** RocA increases the expression of CCL5 in NSCLC cells independent of autophagy inhibition.** A549, H1299, and H1975 cells were exposed to different concentrations (0, 12.5, and 25 nM) of RocA for 24 h, and then the expression of CCL5 at the RNA **(A-C)** and protein **(D-F)** level was analyzed by real-time PCR and ELISA, respectively. **G-I,** A549, H1299, and H1975 cells were exposed to different concentrations (0, 25.0, and 50.0 µM) of CQ for 24 h, and then the expression of CCL5 was analyzed by real-time PCR.** J,** A549, H1299, and H1975 cells were treated with or without 25 nM RocA in the presence or absence of 10µM SP600125 for 24 h, and then the expression of CCL5 was analyzed by real-time PCR. Data were pooled from three independent experiments. **K,** The expressions of ULK1, p62, and LC3 in ULK1 wild-type (WT) and knockout (KO) H1299 cells were detected by Western blotting analysis. Data represented three independent experiments. **L,** ULK1^WT,^ and ULK1^KO^ H1299 cells were exposed to different concentrations (0, 12.5, and 25 nM) of RocA for 24 h, and then the expression of CCL5 was analyzed by real-time PCR. Data were pooled from three independent experiments. *, p < 0.05; **, p < 0.01; ***, p < 0.001; NS, non-statistical significance.

Autophagy inhibition can promote the infiltration of NK cells by up-regulating CCL5 via activating JNK signaling 22, and this led us to investigate whether RocA increased the infiltration of NK cells was due to autophagy inhibition and JNK signaling. A549, H1299, and H1975 cells were exposed to different doses of RocA, and CCL5 was detected by real-time PCR and ELISA. Results showed that RocA clearly increased the expression of CCL5 at the RNA (Fig. 2A-C) and protein levels (Fig. 2D-F) in NSCLC in a dose-dependent manner. Similar experiments were subsequently performed by using autophagy inhibitor CQ and JNK inhibitor SP600125. Results showed that CQ did not significantly increase the expression of CCL5 (Fig. 2G-I), and the up-regulation of CCL5 by RocA could not be reversed by SP600125 (Fig. 2J). Additionally, CQ also did not promote the migration of NK cells to A549, H1299, and H1975 cells (Fig. S1D-G). We have previously found that RocA inhibits autophagy by targeting ULK1. Therefore, the ULK1 was depleted in H1299 cells (Fig. 2K), and the expression of CCL5 induced by RocA was detected. Results showed that RocA still dramatically increased the expression of CCL5 in ULK1^KO^ H1299 cells (Fig 2L). Taken together, these findings demonstrated that RocA could increase the expression of CCL5 in NSCLC cells independent of autophagy inhibition.

### RocA also increases the expression of CXCL10 in NSCLC cells independent of autophagy inhibition

**Figure 3:**
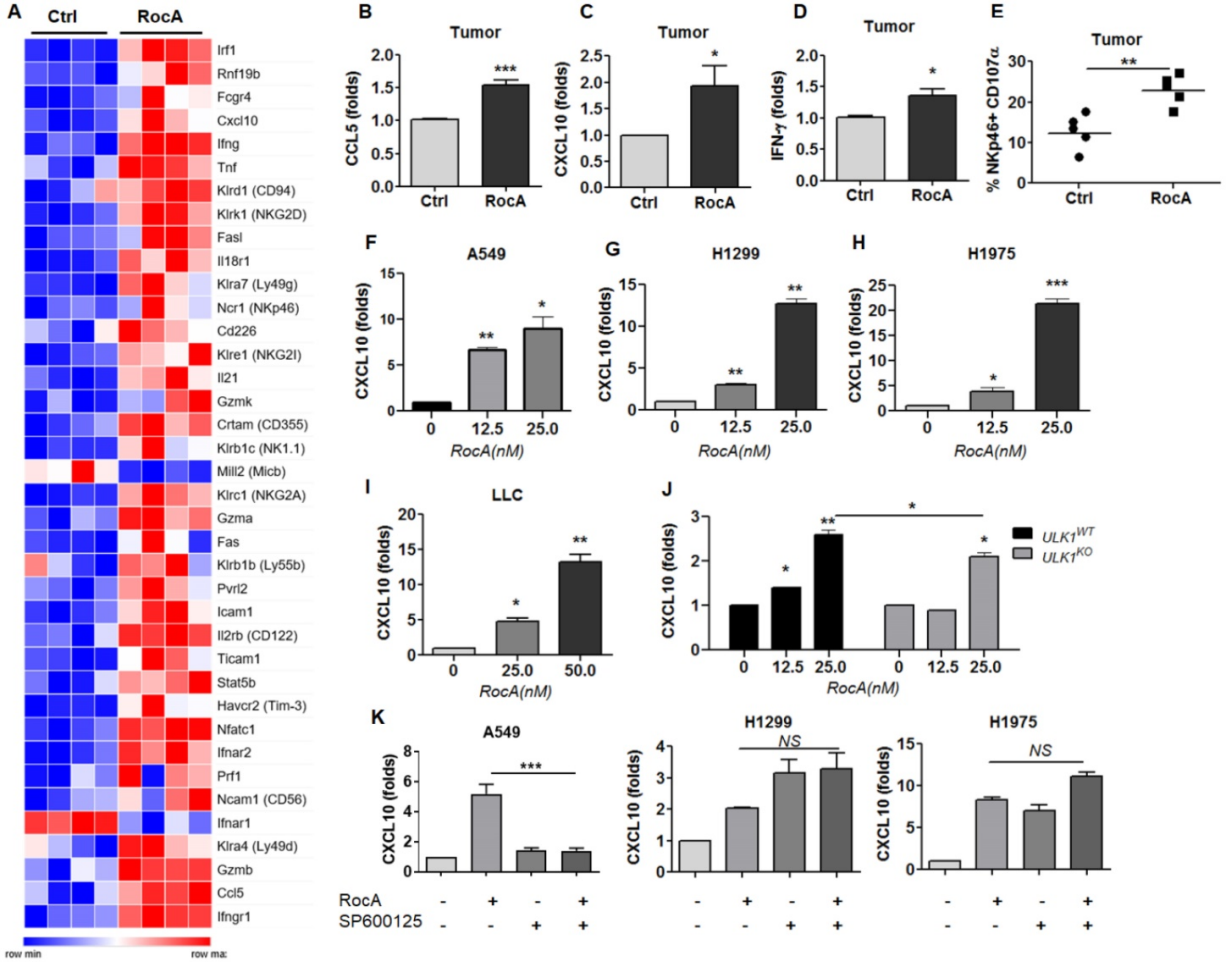
** RocA increases the expression of CXCL10 in NSCLC cells independent of autophagy inhibition. A,** LLC cells were subcutaneously inoculated onto the upper back of C57BL6 mice on day 0, and 1 mg/kg of RocA was administered by i.p. injection every 2 days from day 3. Mice were sacrificed on day 14, and tumors were isolated and used for RNA-Seq. A, DEGs related to NK activation and traffic were richened. **B-D,** The expressions of CCL5, CXCL10, and IFN-γ in tumors were detected by real-time PCR. **E,** The expression of CD107α on the surface of NK cells was analyzed by flow-cytometry. A549 **(F)**, H1299 **(G)**, H1975 **(H)**, and LLC **(I)** cells were exposed to different concentrations (0, 12.5, and 25 nM) of RocA for 24 h, and then the expression of CXCL10 was analyzed by real-time PCR. **J,** ULK1^WT,^ and ULK1^KO^ H1299 cells were exposed to different concentrations (0, 12.5, and 25 nM) of RocA for 24 h, and then the expression of CXCL10 was analyzed by real-time PCR. **K,** A549, H1299, and H1975 cells were treated with or without 25 nM RocA in the presence or absence of 10µM SP600125 for 24 h, and then the expression of CXCL10 was analyzed by real-time PCR. Data were pooled from three independent experiments. *, p < 0.05; **, p < 0.01; ***, p < 0.001; NS, non-statistical significance.

To further define the mechanism, LLC cell-bearing mice were treated with or without RocA for 2 weeks, and tumor tissues were used to analyze differential gene expressions by RNA-Seq. Results showed that the expressions of many activating receptors, such as CRTAM and NKG2D, and effectors, such as GZMB and IFNG, of NK cells, as well as the chemokines CCL5 and CXCL10, were significantly increased by RocA (Fig. 3A). The up-regulation of CCL5 was consistent with the above-mentioned results, suggesting the reliability of RNA-Seq. These findings indicated that RocA could promote NK cell activation and increase the expressions of both CCL5 and CXCL10 in LLC tumors, suggesting that RocA-induced infiltration of NK cells could be attributed to the up-regulation of both CCL5 and CXCL10. Subsequently, the up-regulation of CCL5, CXCL10, and IFN-γ in LLC tumor tissues was confirmed by real-time PCR (Fig. 3B-D). The activation of NK cells was also confirmed by the expression of CD107α (Fig. 3E). In parallel, the expression of CXCL10 by RocA in NSCLC cells was then investigated. Results showed that RocA significantly increased the expression of CXCL10 inA549 (Fig. 3F), H1299 (Fig. 3G), H1975 (Fig. 3H), and LLC (Fig. 3I) cells in a dose-dependent manner. Moreover, RocA still significantly increased the expression of CXCL10 in ULK1^KO^ H1299 cells (Fig. 3J), and the up-regulation of CXCL10 by RocA was not be reversed by SP600125 (Fig. 3K). Taken together, these results demonstrated that except for CCL5, RocA also increased the expression of CXCL10 in NSCLC cells independent of autophagy inhibition.

### RocA increases the expressions of CCL5 and CXCL10 depending on TBK1 and STING

**Figure 4:**
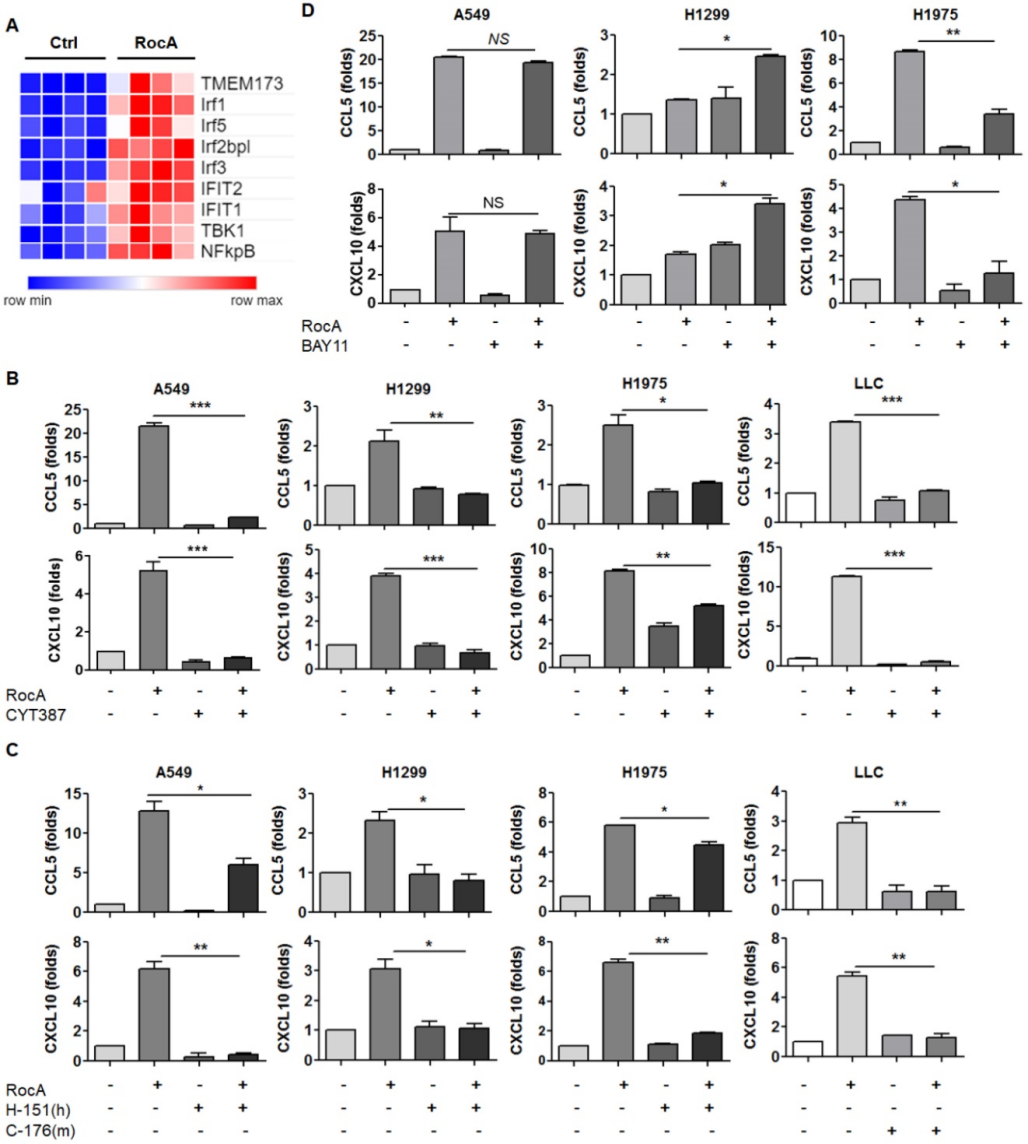
** RocA increases the expressions of CCL5 and CXCL10 depending on TBK1 and STING. A,** DEGs related to the cGAS-STING signaling pathway were richened. A549, H1299, H1975, and LLC cells were treated with or without 25 nM of RocA in the presence or absence of 5 µM CYT387 **(B)**, 5 µM H-151 or 5 µM C-176 **(C)**, or 10 µM BAY11 for 24 h, and then the expressions of CCL5 and CXCL10 were analyzed by real-time PCR. Data were pooled from three independent experiments. *, p < 0.05; **, p < 0.01; ***, p < 0.001.

**Figure 5:**
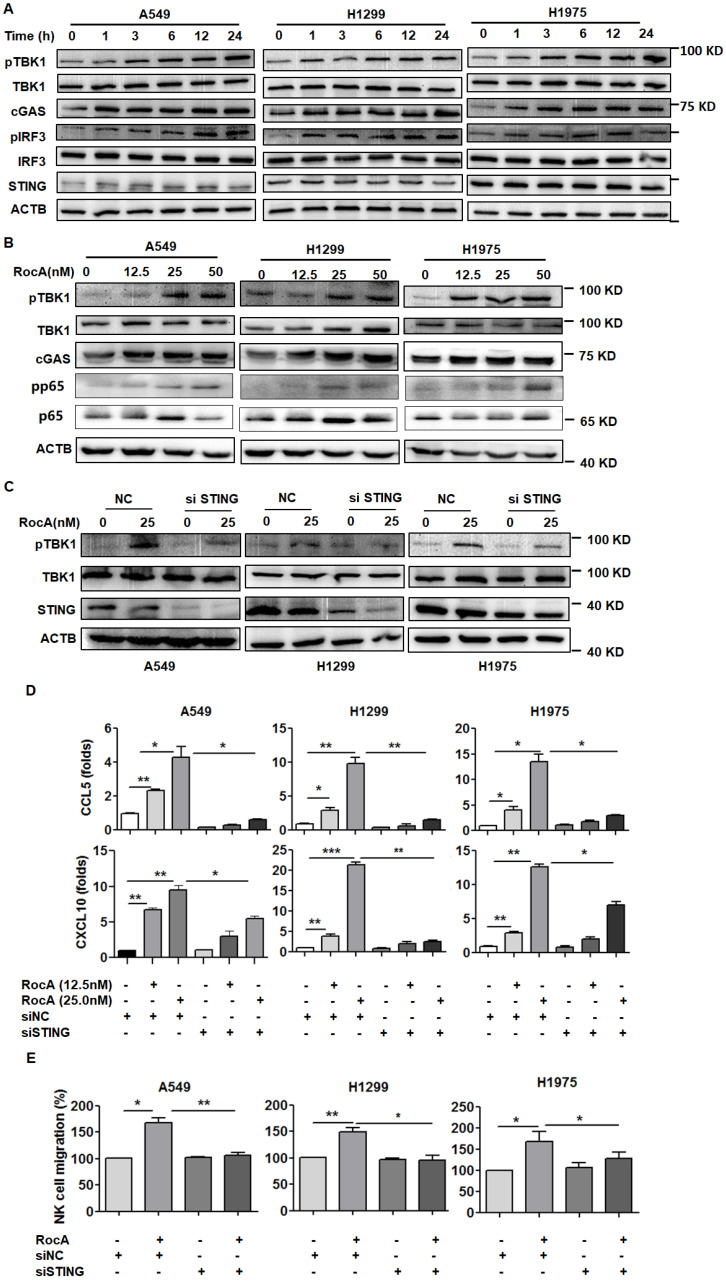
** RocA activates the cGAS-STING signaling pathway and increases the expressions of CCL5 and CXCL10 depending on such pathway. A,** A549, H1299, and H1975 cells were exposed to 25 nM of RocA for different durations (0, 1, 3, 6, 12, and 24 h), and then the expressions of cGAS, STING, pTBK1, TBK1, pIRF3, and IRF3 were detected by Western blotting analysis. **B,** A549, H1299, and H1975 cells were exposed to different concentrations (0, 12.5, 25, and 50 nM) of RocA for 24 h, and then the expressions of cGAS, pTBK1, TBK1, p65, and pp65 were detected by Western blotting analysis. **C,** A549, H1299, and H1975 cells were transfected with STING siRNA or negative control (NC) for 24 h and then exposed to 25 nM of RocA for 24 h, followed by the detection of STING, pTBK1, and TBK1 by Western blotting analysis. Data represented three independent experiments. **E,** A549, H1299, and H1975 cells were transfected with STING siRNA or NC for 24 h and then exposed to different concentrations (0, 12.5, and 25 nM) of RocA for 24 h, followed by the detection of CCL5 and CXCL10 by real-time PCR. **F,** A549, H1299, and H1975 cells were transfected with STING siRNA or NC for 24 h and then exposed to 25 nM of RocA for 24 h, followed by the analysis of NK cell migration. Data were pooled from three independent experiments. *, p < 0.05; **, p < 0.01; ***, p < 0.001.

The above-mentioned results demonstrated that RocA increased the expressions of CCL5 and CXCL10 in NSCLC cells independent of autophagy/ULK1 inhibition. To further investigate the mechanism of action, RNA-Seq data were further analyzed, and the results showed that the expressions of STING (TMEM173), TBK1, IRF3 and NF-κB were significantly increased by RocA (Fig. 4A). These findings suggested that RocA might increase the expressions of CCL5 and CXCL10 by activating the STING signaling pathway. To confirm this issue, the expressions of STING, TBK1, IRF3, and NF-κB were determined by real-time PCR. Results showed that RocA did not increase the expressions of STING, TBK1, IRF3, and NF-κB at the mRNA level in all three cell lines (Fig. S2), while RocA significantly increased the expression of pp65 in a dose-dependent manner (Fig. 5B). The TBK1 inhibitor CYT387, human STING inhibitor H-151, mouse STING inhibitor C-176, and NF-κB inhibitor BAY11 were then used to inhibit TBK1, STING, and NF-κB, respectively. Results showed that the up-regulation of CCL5 and CXCL10 by RocA was dramatically reversed by inhibitions of either TBK1 (Fig. 4B) or STING (Fig. 4C), but not by NF-κB inhibition (Fig. 4D). Taken together, these results suggested that RocA increased the expressions of CCL5 and CXCL10 in NSCLC cells depending on TBK1 and STING.

### RocA activates the cGAS-STING signaling pathway to induce the expressions of CCL5 and CXCL10 in NSCLC cells

The above-mentioned results suggested that RocA could activate the STING signaling pathway. To further confirm this issue, A549, H1299, and H1975 cells were then treated with RocA, and proteins in the cGAS-STING signaling pathway were detected. Results showed that RocA significantly increased the expressions of cGAS, pTBK1, and pIRF3 in a time- and dose-dependent manner (Fig. 5A-B & Fig. S3). The STING was then knock-downed by siRNA, and RocA-induced up-regulation of pTBK1 was dramatically reduced in STING knockdown cells (Fig. 5C). Furthermore, the knockdown of STING significantly reduced the up-regulation of CCL5 and CXCL10 (Fig. 5D) and the migration of NK cells (Fig. 5E) induced by RocA. These results indicated that RocA activated cGAS-STING signaling in NSCLC cells, leading to the up-regulation of CCL5 and CXCL10 and enhanced infiltration of NK cells.

### RocA enhances infiltration and antitumor immunity of NK cells depending on STING

**Figure 6:**
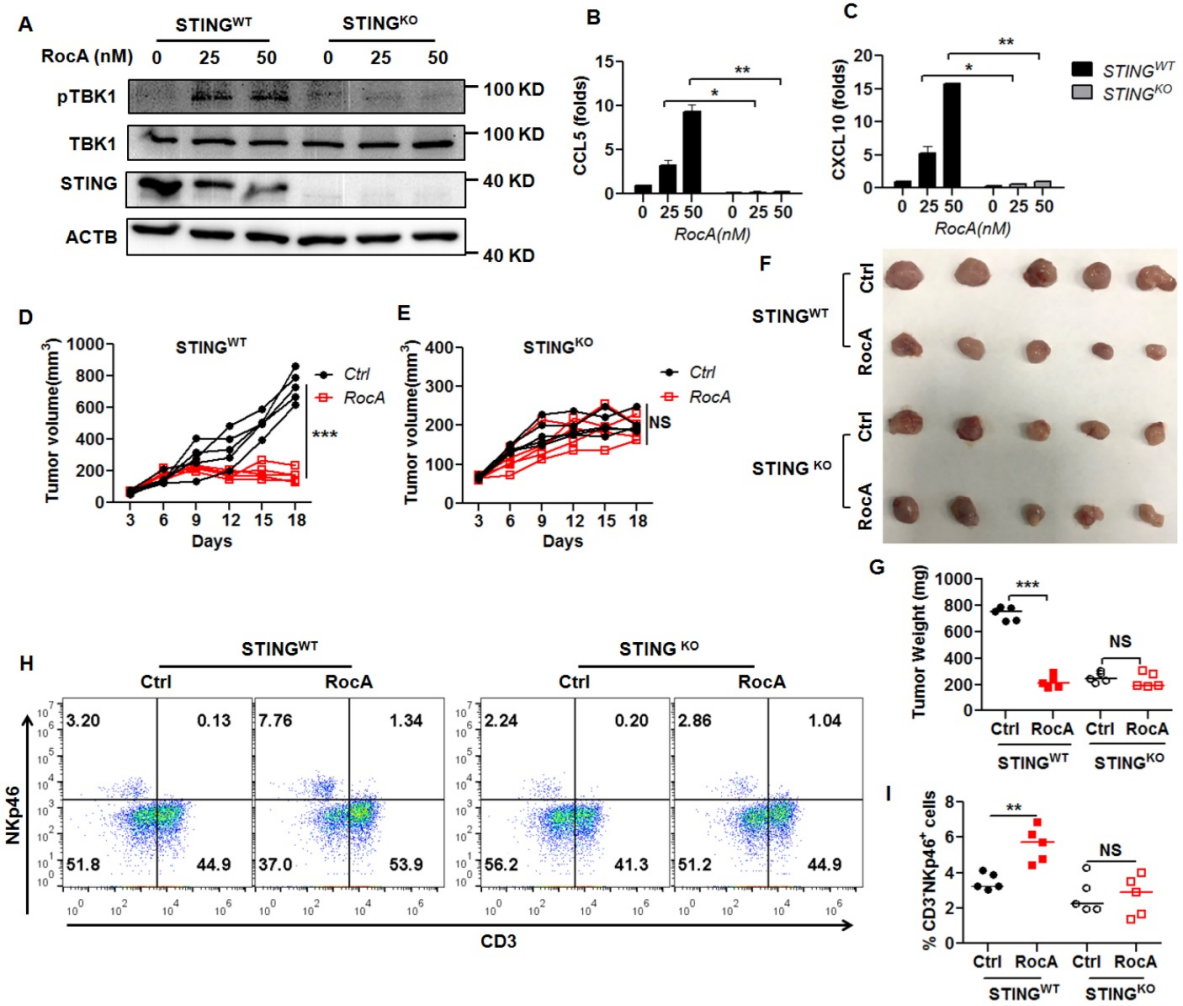
** NK cell infiltration and tumor regression by RocA depend on STING. A,** The expressions of STING, pTBK1, and TBK1 in STING^WT^ and STING^KO^ LLC cells were detected by Western blotting analysis. Data represented three independent experiments. STING^WT^ and STING^KO^ LLC cells were exposed to different concentrations (0, 12.5, and 25 nM) of RocA for 24 h, and then the expressions of CCL5 **(B)** and CXCL10 **(C)** were analyzed by real-time PCR. Data were pooled from three independent experiments. STING^WT^ and STING^KO^ LLC cells were subcutaneously inoculated onto the upper back of C57BL6 mice on day 0, and 1 mg/kg of RocA was administered by i.p. injection every 2 days from day 3. Tumor size was measured every 2 days **(D, E)**. Mice were sacrificed on day 18, and tumors were excised, photographed **(F)**, weighed **(G)**, and used to detect the proportions of NK cells **(H-I)**. Data represented three independent experiments. *, p < 0.05; **, p < 0.01; ***, p < 0.001; NS, non-statistical significance.

Since RocA could activate the cGAS-STING signaling pathway, leading to the increased infiltration of NK cells, we next determined whether RocA increased infiltration and antitumor immunity of NK cells depending on STING signaling. Therefore, STING was depleted in LLC cells. Results demonstrated that RocA no longer increased the expressions of pTBK1 (Fig. 6A), CCL5 (Fig. 6B), and CXCL10 (Fig. 6C) in STING^KO^ LLC cells. Furthermore, RocA did not inhibit the tumor growth of STING^KO^ LLC cells (Fig. 6D-G), and did not increase the proportion of NK cells in STING^KO^ LLC tumors (Fig. 6H-I). These results illuminated that RocA enhanced the infiltration and antitumor immunity of NK cells depending on STING signaling.

### RocA damages mtDNA and promotes mtDNA cytoplasmic release

**Figure 7:**
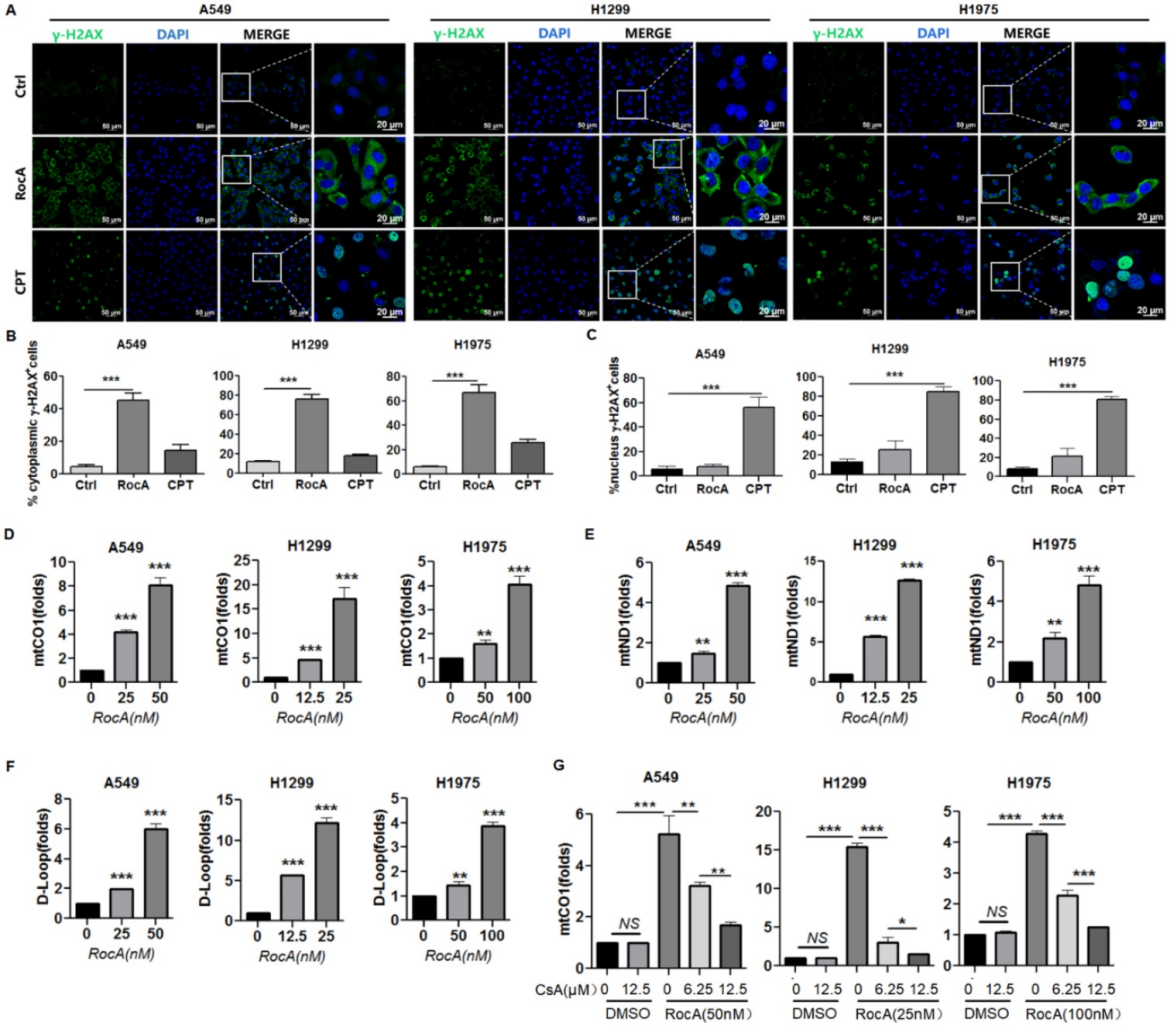
** RocA damages mtDNA and promotes the cytoplasmic release of mtDNA. A549, H1299, and H1975 cells were exposed to 25 nM of RocA or 5 µM of CPT for 24 h. A,** The cells were stained with anti-γH2AX antibody and DAPI, and observed under laser confocal microscopy. **B,** The proportions of γH2AX^+^ cells in the cytoplasm. **C,** The proportions of γH2AX^+^ cells in the nucleus. **D-F,** A549, H1299, and H1975 cells were exposed to different concentrations (0, 12.5, and 25 nM) of RocA for 24 h, and then the expressions of CO1, ND1, and D-Loop in the cytoplasm were detected by real-time PCR. **G,** A549, H1299, and H1975 cells were exposed to different concentrations (0, 12.5, and 25 nM) of RocA or different concentrations (0, 6.25, and 12.5 µM) of CsA for 24 h, and then the expression of CO1 in the cytoplasm was detected by real-time PCR. Data represented three independent experiments. *, p < 0.05; **, p < 0.01; ***, p < 0.001; NS, non-statistical significance.

cGAS is a cytosolic DNA sensor that recognizes and binds with double-stranded DNA (dsDNA) in the cytoplasm. The DNA binding can activate cGAS, which in turn stimulates STING to trigger interferon signaling 23. Endogenous DNA sources that trigger STING include damaged nuclear DNA (nDNA) and mtDNA. We showed that RocA could activate cGAS-STING signaling. Therefore, we investigated the damage of RocA to nDNA and mtDNA. A549, H1299, and H1975 cells were treated with RocA, or CPT, which can cause dsDNA damage during the S-phase of cell replication, and then stained the cells with an antibody against γ-H2AX, which is a sensitive marker of dsDNA damage. The increased γ-H2AX foci were observed in the cytoplasm in RocA-treated NSCLC cells, not in the nucleus. Inversely, the increased γ-H2AX foci were observed in the nucleus in CPT-treated NSCLC cells, not in the cytoplasm (Fig. 7A-C). This finding indicated that RocA damaged mtDNA and had no clear damage in nDNA, suggesting that the endogenous DNA triggering STING by RocA was attributed to damaged mtDNA. Subsequently, the mtDNA cytoplasmic release was detected. Results showed that RocA significantly increased the expressions of mitochondrial CO1, ND1, and D-Loop in the cytoplasm in a dose-dependent manner (Fig. 7D-F). Mitochondrial permeability transition pore (mPTP) has been recently found to be critical for mtDNA release, and the mPTP inhibitor CsA can efficiently prevent mtDNA leakage into the cytoplasm 24. Therefore, we investigated the effect of CsA and found that CsA significantly reduced the cytoplasmic release of mtDNA induced by RocA in a dose-dependent manner (Fig. 7G). Taken together, these results demonstrated that RocA damaged mtDNA and promoted the cytoplasmic release of mtDNA, which in turn stimulated the activation of cGAS-STING signaling.

## Discussion

Immune cell infiltration and homing to the tumor are critical for cancer immunotherapy. In the current study, we found that RocA damaged mtDNA and then promoted the cytoplasmic release of mtDNA, which in turn activated cGAS-STING signaling, resulting in enhanced infiltration and antitumor immunity of NK cells. This finding demonstrated that RocA was a novel agonist of cGAS-STING by targeting mtDNA, and it had a promising potential in cancer immunotherapy.

Targeting autophagy has been shown to allow the trafficking of cytotoxic NK cells into the tumor tissue by a mechanism involving the activation of the JNK signaling pathway in melanoma 25. Therefore, as a cutting-edge approach, autophagy inhibition can promote NK cell infiltration and improve the antitumor immunity of NK cells 22. The highly conserved serine/threonine kinase ULK1 is the human homolog of the Saccharomyces cerevisiae autophagy-related protein kinase Atg1, and it plays a crucial role in the initiation of autophagy 26. In our previous study, we have found that RocA inhibits autophagy by targeting ULK1, suggesting that RocA promotes the infiltration of NK cells by autophagy inhibition. However, we found that autophagy inhibition by CQ or ULK1 depletion did not increase the expression of CCL5 or CXCL10 in NSCLC cells, suggesting that autophagy inhibition could not promote the infiltration of NK cells into NSCLC tumor. This finding was inconsistent with the previous report in melanoma, suggesting that autophagy inhibition for NK cell infiltration was selective in various tumors.

The cGAS-STING signaling plays an important role in innate antitumor immunity, and it is an attractive anti-cancer immunotherapeutic drug target 27. Cytoplasmic self-DNA can be recognized by cGAS, leading to activation of STING, which is an essential signal adaptor mediating cytosolic DNA-induced innate immune responses. In the present study, we found that RocA could activate the cGAS-STING signaling pathway by targeting mtDNA, which attracted NK cell infiltration and homing to NSCLC tumors in a STING-dependent manner. These findings suggested that RocA was a potent cGAS-STING agonist and played a critical role in innate antitumor immunity.

Mitochondria are involved in cancer progression and emerging as a promising therapeutic target for cancer therapy 28. mtDNA can be released from the mitochondria into the cytoplasm and play a key role in inflammation and immunity 29, suggesting that mtDNA is a promising target for cancer therapy. For example, ATM (ataxia telangiectasia mutated) inhibition promotes mtDNA leakage into the cytoplasm to activate cGAS-STING signaling and enhance lymphocyte infiltration into the tumor microenvironment 30. Radiation therapy enables exposure of mtDNA to the cytosol, triggering cGAS-STING driven type I IFN to elicit anticancer immunity 31. In our current study, we found that RocA damaged mtDNA to promote mtDNA leakage into the cytoplasm, and elicited STING-dependent antitumor immunity of NK cells. Our finding supported that mtDNA was a potential target to enhance innate antitumor immunity, suggesting that RocA was a novel and promising reagent for targeting mtDNA.

Collectively, we demonstrated that RocA specifically damaged mtDNA, then promoted mtDNA leakage into the cytoplasm and activated the cGAS-STING signaling pathway, leading to increased infiltration of NK cells and enhanced antitumor immunity in NSCLC. We, for the first time, showed that RocA promoted STING-dependent infiltration and antitumor immunity of NK cells by targeting mtDNA, suggesting that RocA had a promising potential in cancer immunotherapy as a potent agonist of cGAS-STING.

## Supplementary Material

Supplementary figures.

## References

[1] Crinier A, Narni-Mancinelli E, Ugolini S, Vivier E (2020). SnapShot: Natural Killer Cells. Cell.

[2] Tarazona R, Lopez-Sejas N, Guerrero B, Hassouneh F, Valhondo I, Pera A (2020). Current progress in NK cell biology and NK cell-based cancer immunotherapy. Cancer Immunol Immunother.

[3] Halama N, Braun M, Kahlert C, Spille A, Quack C, Rahbari N (2011). Natural killer cells are scarce in colorectal carcinoma tissue despite high levels of chemokines and cytokines. Clin Cancer Res.

[4] Kremer V, Ligtenberg MA, Zendehdel R, Seitz C, Duivenvoorden A, Wennerberg E (2017). Genetic engineering of human NK cells to express CXCR2 improves migration to renal cell carcinoma. J Immunother Cancer.

[5] Nayyar G, Chu Y, Cairo MS (2019). Overcoming Resistance to Natural Killer Cell Based Immunotherapies for Solid Tumors. Front Oncol.

[6] Chow MT, Luster AD (2014). Chemokines in cancer. Cancer Immunol Res.

[7] Wennerberg E, Kremer V, Childs R, Lundqvist A (2015). CXCL10-induced migration of adoptively transferred human natural killer cells toward solid tumors causes regression of tumor growth *in vivo*. Cancer Immunol Immunother.

[8] Mgrditchian T, Arakelian T, Paggetti J, Noman MZ, Viry E, Moussay E (2017). Targeting autophagy inhibits melanoma growth by enhancing NK cells infiltration in a CCL5-dependent manner. Proc Natl Acad Sci U S A.

[9] Porporato PE, Filigheddu N, Pedro JMB, Kroemer G, Galluzzi L (2018). Mitochondrial metabolism and cancer. Cell Res.

[10] Denisenko TV, Gorbunova AS, Zhivotovsky B (2020). Mitochondrial Involvement in Migration, Invasion and Metastasis. Front Cell Dev Biol.

[11] Zong WX, Rabinowitz JD, White E (2016). Mitochondria and Cancer. Mol Cell.

[12] Klein K, He K, Younes AI, Barsoumian HB, Chen D, Ozgen T (2020). Role of Mitochondria in Cancer Immune Evasion and Potential Therapeutic Approaches. Front Immunol.

[13] Dong L, Gopalan V, Holland O, Neuzil J (2020). Mitocans Revisited: Mitochondrial Targeting as Efficient Anti-Cancer Therapy. Int J Mol Sci.

[14] Giaisi M, Kohler R, Fulda S, Krammer PH, Li-Weber M (2012). Rocaglamide and a XIAP inhibitor cooperatively sensitize TRAIL-mediated apoptosis in Hodgkin's lymphomas. Int J Cancer.

[15] Bleumink M, Kohler R, Giaisi M, Proksch P, Krammer PH, Li-Weber M (2011). Rocaglamide breaks TRAIL resistance in HTLV-1-associated adult T-cell leukemia/lymphoma by translational suppression of c-FLIP expression. Cell Death Differ.

[16] Neumann J, Boerries M, Kohler R, Giaisi M, Krammer PH, Busch H (2014). The natural anticancer compound rocaglamide selectively inhibits the G1-S-phase transition in cancer cells through the ATM/ATR-mediated Chk1/2 cell cycle checkpoints. Int J Cancer.

[17] Yurugi H, Marini F, Weber C, David K, Zhao Q, Binder H (2017). Targeting prohibitins with chemical ligands inhibits KRAS-mediated lung tumours. Oncogene.

[18] Luan Z, He Y, He F, Chen Z (2015). Rocaglamide overcomes tumor necrosis factor-related apoptosis-inducing ligand resistance in hepatocellular carcinoma cells by attenuating the inhibition of caspase-8 through cellular FLICE-like-inhibitory protein downregulation. Mol Med Rep.

[19] Nalli AD, Brown LE, Thomas CL, Sayers TJ, Porco JA Jr, Henrich CJ (2018). Sensitization of renal carcinoma cells to TRAIL-induced apoptosis by rocaglamide and analogs. Sci Rep.

[20] Yao C, Ni Z, Gong C, Zhu X, Wang L, Xu Z (2018). Rocaglamide enhances NK cell-mediated killing of non-small cell lung cancer cells by inhibiting autophagy. Autophagy.

[21] Wang X, Lee DA, Wang Y, Wang L, Yao Y, Lin Z (2013). Membrane-bound interleukin-21 and CD137 ligand induce functional human natural killer cells from peripheral blood mononuclear cells through STAT-3 activation. Clin Exp Immunol.

[22] Noman MZ, Paggetti J, Moussay E, Berchem G, Janji B (2018). Driving Natural Killer cells toward the melanoma tumor battlefield: Autophagy as a valuable therapeutic target. Oncoimmunology.

[23] Ma Z, Damania B (2016). The cGAS-STING Defense Pathway and Its Counteraction by Viruses. Cell Host Microbe.

[24] Yu CH, Davidson S, Harapas CR, Hilton JB, Mlodzianoski MJ, Laohamonthonkul P (2020). TDP-43 Triggers Mitochondrial DNA Release via mPTP to Activate cGAS/STING in ALS. Cell.

[25] Noman MZ, Berchem G, Janji B (2018). Targeting autophagy blocks melanoma growth by bringing natural killer cells to the tumor battlefield. Autophagy.

[26] Chan EY, Kir S, Tooze SA (2007). siRNA screening of the kinome identifies ULK1 as a multidomain modulator of autophagy. J Biol Chem.

[27] Zhang X, Bai XC, Chen ZJ (2020). Structures and Mechanisms in the cGAS-STING Innate Immunity Pathway. Immunity.

[28] Dhanasekaran S, Venugopal D, Al-Dayan N, Ravinayagam V, Mohammed AA (2020). Emerging insights into mitochondria-specific targeting and drug delivering strategies: Recent milestones and therapeutic implications. Saudi J Biol Sci.

[29] Riley JS, Tait SW (2020). Mitochondrial DNA in inflammation and immunity. EMBO Rep.

[30] Hu M, Zhou M, Bao X, Pan D, Jiao M, Liu X (2021). ATM inhibition enhances cancer immunotherapy by promoting mtDNA leakage and cGAS/STING activation. J Clin Invest.

[31] Yamazaki T, Galluzzi L (2020). Mitochondrial control of innate immune signaling by irradiated cancer cells. Oncoimmunology.

