# Supplementary material for: Rocaglamide promotes the infiltration and antitumor immunity of NK cells by activating cGAS-STING signaling in non-small cell lung cancer
Source: Int J Biol Sci. 2022 Jan 1;18(2):585–98. doi: 10.7150/ijbs.65019 (PMC8741839; doi:10.7150/ijbs.65019)
Supplement: Supplementary file 1 — Supplementary figures. [file ijbsv18p0585s1.pdf]

Supplementary Figures and Figure Legends

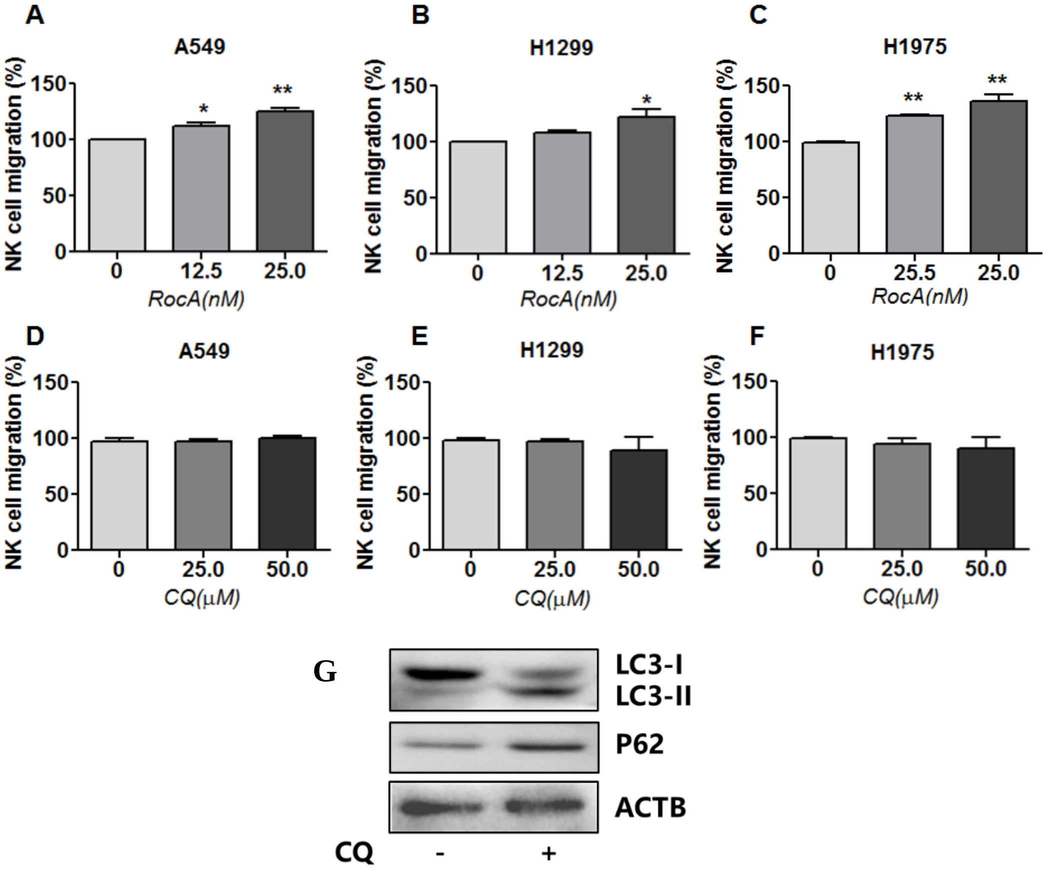

Figure S1. The effects of RocA and CQ on the migration of NK cells and the inhibition of CQ on autophagy. A549, H1299, and H1975 cells were exposed to different concentrations (0, 12.5, and 25 nM) of RocA (A-C), or different concentrations (0, 25.0, and 50.0 μM) of CQ (D-F) for 24 h, and then seeded into the bottom chamber. NK cells were seeded into the upper compartment. After 2 h, NK cells in the bottom chamber were analyzed by flow cytometry. Data were pooled from three independent experiments. *, $p < 0.05$; **, $p < 0.01$. G, H1299 cells were exposed to 50 μM CQ for 4 h, then lysed and analyzed to detect the LC3 and p62 proteins. Independent experiments were repeated twice.

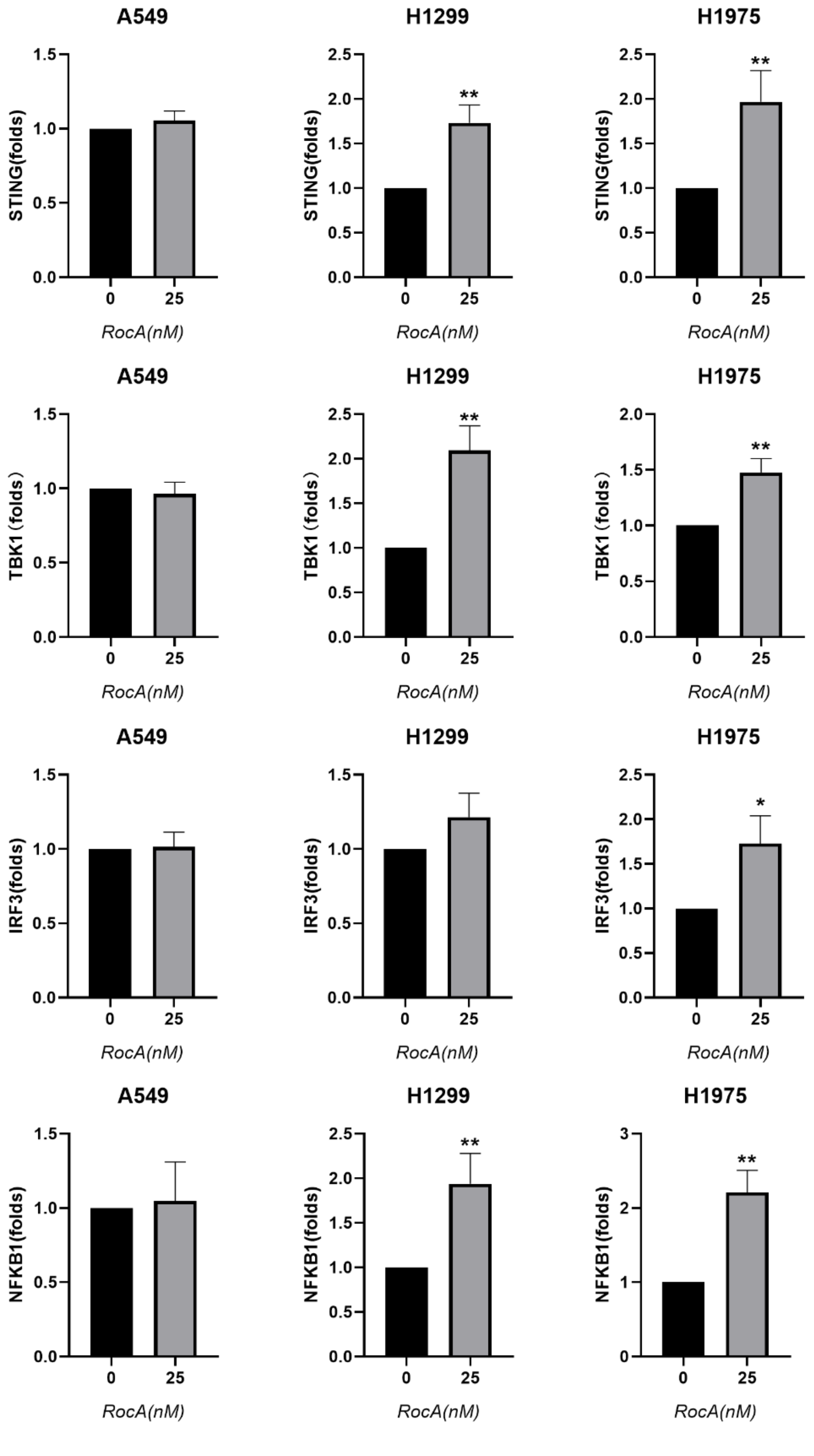

Figure S2. The expressions of STING, TBK1, IRF3, and NF-κB by RocA. A549, H1299 and H1975 cells were exposed to 0 or 25nM of RocA for 24h. The expressions of STING, TBK1, IRF3, and NF-κB were detected by real-time PCR. Results were normalized to 18S.

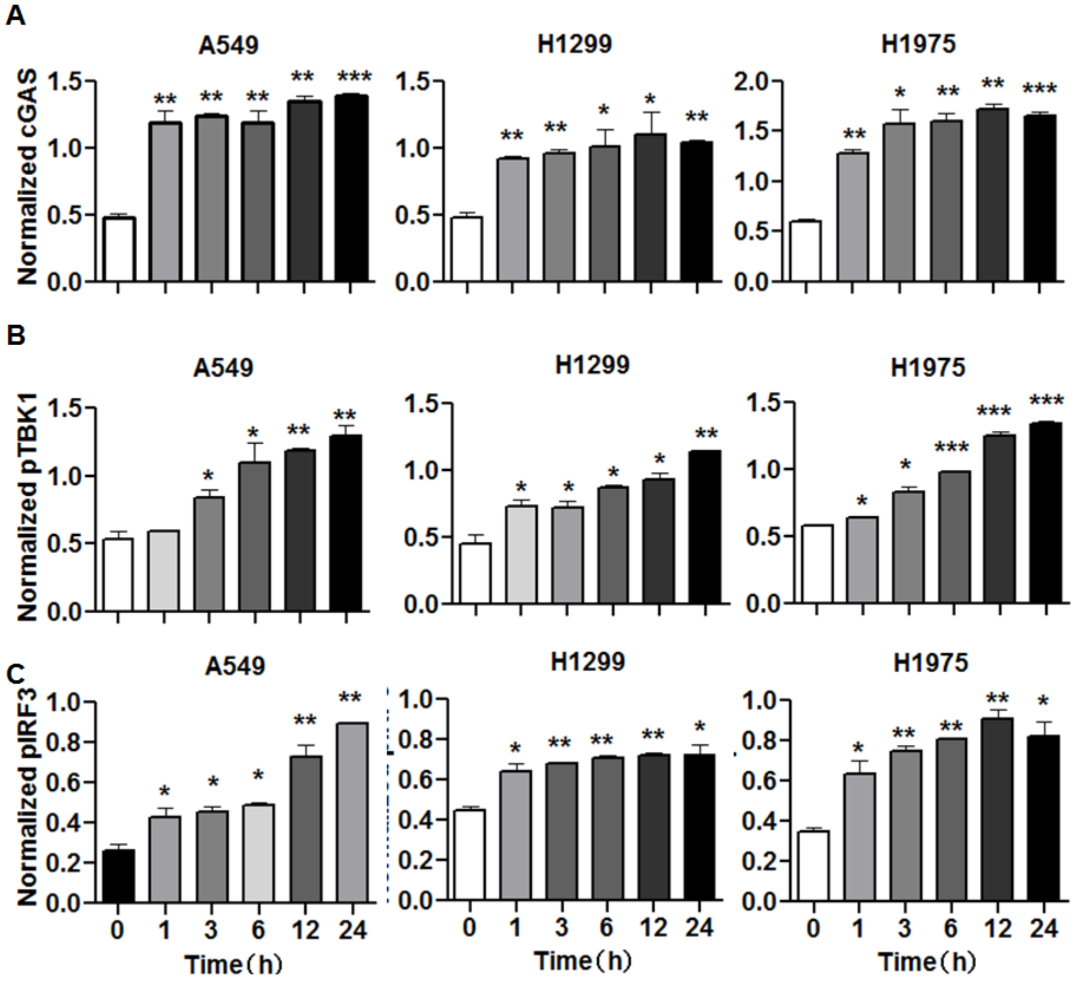

Figure S3. RocA activates the cGAS-STING signaling pathway in NSCLC cells. The expressions of cGAS (A), pTBK1 (B), and pIRF3 (C) at the protein level in A549, H1299, and H1975 cells were analyzed. Data were from Figure 5A. *, $p < 0.05$; **, $p < 0.01$; ***, $p < 0.001$.
